# Using DHS and MICS data to complement or replace NGO baseline health data: an exploratory study

**DOI:** 10.12688/f1000research.47618.1

**Published:** 2021-02-04

**Authors:** Peter R. Berti, Milena Nardocci, Minh Hung Tran, Malek Batal, Rebecca Brodmann, Nicolas Greliche, Naomi M. Saville

**Affiliations:** 1 HealthBridge Foundation of Canada, Ottawa, Canada; 2 TRANSNUT, Université de Montréal, Montreal, Canada; 3 Center for Creative Initiatives in Health and Population, Hanoi, Vietnam; 4 Statistics for Sustainable Development, Reading, UK; 5 University College London Institute for Global Health, London, UK

**Keywords:** DHS, MICS, surveys, maternal and child health

## Abstract

**Background:** Non-government organizations (NGOs) spend substantial time and resources collecting baseline data in order to plan and implement health interventions with marginalized populations. Typically interviews with households, often mothers, take over an hour, placing a burden on the respondents. Meanwhile, estimates of numerous health and social indicators in many countries already exist in publicly available datasets, such as the Demographic and Health Surveys (DHS) and the Multiple Indicator Cluster Surveys (MICS), and it is worth considering whether these could serve as estimates of baseline conditions. The objective of this study was to compare indicator estimates from non-governmental organizations (NGO) health projects’ baseline reports with estimates calculated using the Demographic and Health Surveys (DHS) or the Multiple Indicator Cluster Surveys (MICS), matching for location, year, and season of data collection.

**Methods:** We extracted estimates of 129 indicators from 46 NGO baseline reports, 25 DHS datasets and three MICS datasets, generating 1,996 pairs of matched DHS/MICS and NGO indicators. We subtracted NGO from DHS/MICS estimates to yield difference and absolute difference, exploring differences by indicator. We partitioned variance of the differences by geographical level, year, and season using ANOVA.

**Results: **Differences between NGO and DHS/MICS estimates were large for many indicators but 33% fell within 5% of one another. Differences were smaller for indicators with prevalence <15% or >85%. Difference between estimates increased with increasing year and geographical level differences. However, <1% of the variance of the differences was explained by year, geographical level, and season.

**Conclusions: **There are situations where publicly available data could complement NGO baseline survey data, most importantly when the NGO has tolerance for estimates of low or unknown accuracy.

## Introduction

Non-government and civil society organizations spend substantial time and resources collecting baseline data in order to plan and implement health interventions with marginalized populations, and to measure the impact of those interventions ( Data for Impact, 2019). Typical methods involve baseline and endline household surveys, where the household residents are interviewed and asked a hundred or more questions about asset ownership, mother and child health, diet, health system access, and other topics of interest. The costs of these surveys vary depending on design, methods, sample size, survey length, and local context ( Data for Impact, 2019), but in the authors’ experience tens of thousands of dollars is typical, and in some cases, much more. Depending on the number and nature of questions, interviews can be over an hour long, placing a burden on the respondents. In addition, the accuracy of the indicator estimates in NGO-led surveys may be insufficient for project design and monitoring purposes, due to relatively small sample sizes and the inherent high variability of the indicators of interest.

Meanwhile, estimates of numerous health and social indicators in many countries already exist in publicly available datasets, such as the Demographic and Health Surveys (DHS), supported by USAID ( U.S. Agency for International Development, 2018), and the Multiple Indicator Cluster Surveys (MICS), supported by UNICEF ( UNICEF, 2020), and it is worth considering whether these could serve as estimates of baseline conditions. DHS/MICS provide standardized data collected using rigorous methods and large sample sizes, and datasets are available on request for free. They are designed to be representative at the national, regional and provincial level (but rarely at lower levels, such as district and village, where NGOs are working), and probably exclude homeless, institutionalized and nomadic populations ( Carr-Hill, 2013). DHS/MICS are collected every three to ten years so there may up to ten-years gap between DHS/MICS data collection and the baseline conditions that the NGO wants characterized. Although some indicators’ descriptions have been modified and improved over time, caution is taken to ensure that data are directly comparable across countries, regions and years ( Hancioglu & Arnold, 2013; UNICEF, 2020; U.S. Agency for International Development, 2018). DHS/MICS surveys are adapted to specific country needs and are conducted by well-trained interviewers who have access to tools and guidelines for quality assurance throughout ( UNICEF, 2020; U.S. Agency for International Development, 2018).

Using publicly available data to complement or replace NGOs’ primary data collection for project baseline measures and project monitoring would save valuable resources, reducing the burden on data collectors and respondents alike. A few studies have compared estimates between DHS/MICS and NGO surveys. One found that they provided very different estimates of electricity and water access in Kenya, Tanzania, and Uganda ( Carr-Hill, 2017), and a second found that DHS and a NGO-led survey provided similar estimates of several maternal and child health estimates in Rwanda ( Langston *et al.*, 2015). Other studies found that estimates of the market share of faith-based health care providers by DHS and NGO surveys in sub-Saharan Africa were within 5 to 50% of each other ( Wodon *et al.*, 2012), and the confidence intervals for the difference between Lot Quality Assurance Sampling (LQAS) and DHS district-level estimates were within +/-10% for 15 of 37 health indicators ( Anoke *et al.*, 2015). Therefore, no consensus exists on the potential for DHS/MICS to substitute NGO surveys.

We hypothesized that publicly available data can provide estimates of baseline conditions similar to those reported in NGO baseline reports when matched as closely as possible for location, year, and season of data collection. We tested this hypothesis by comparing indicator estimates from NGO reports with estimates calculated using DHS/MICS.

## Methods

### Data from NGO baseline reports

We collected and retained a sample of 46 NGO baseline reports through a combination of internet search and personal contacts with Canadian and Vietnamese NGOs using the following selection criteria:

i) household survey (n>100) which used valid methods and representative sampling to generate point estimates of maternal, newborn and child health indicators;
ii) conduced between 2005 and 2019;
iii) in a low- or middle-income country.

The baseline reports from NGOs working on maternal, newborn and child health covered 23 countries spanning South Asia (Bangladesh, India, Pakistan), Africa (Burkina Faso, Ethiopia, Ghana, Kenya, Liberia, Malawi, Mali, Mozambique, Nigeria, Senegal, South Sudan, Tanzania, Zambia), South/Central America (Bolivia, Honduras), the Caribbean (Haiti), and SE Asia (Laos, Myanmar, Philippines, Vietnam) ( Table 1) ( Berti, 2021). From the reports, we extracted: country name, NGO name, dates of data collection, population of study, inclusion/exclusion criteria, indicator name and definition, sample size (total and *n* for each indicator), and the indicator estimate (percentage and standard deviation (SD) if available).

**Table 1.** NGOs’ baseline report and matched data from DHS/MICS.

	NGO					DHS/MICS				
Country	Source	Year	Sample size	Geographical location	Level *	Source	Year	Sample size	Geographical location	Level *
Bangladesh	A&T	2010	4,400	Divisions of Dhaka, Chittagong, Rajshahi, Khulna, Barisal, and Sylhet	3rd	DHS	2007	4,923	Divisions of Dhaka, Chittagong, Rajshahi, Khulna, Barisal, and Sylhet	3rd
Bangladesh	PLAN (BORN)	2016	900	Upazilas of Pirgachha, Pirganj, Mithapukur, Kaunia and Gangachara (rural area only) and district of Rangpur	1st, 2nd	DHS	2014	265	Division of Rangpur (rural area only)	3rd
Bangladesh	NIMS	2017	963	Divisions of Dhaka, Chittagong, Khulna, Rajshahi, Sylhet, Barisal	3rd	DHS	2014	409	Divisions of Dhaka, Chittagong, Khulna, Rajshahi, Sylhet, Barisal	3rd
Bangladesh	PLAN (SHOW)	2016	864	Districts of Barisal, Chittagong and Rangpur	2nd	DHS	2014	1,314	Divisions of Barisal, Chittagong and Rangpur	3rd
Bangladesh	WV	2018	33,600	National and by districts (Barisal, Pirojpur, Bandarban, Chittagong, Comilla, Dhaka, Gazipur, Gopalganj, Tangail, Bagerhat, Satkhira, Mymensingh, Netrakona, Sherpur, Naogaon, Rajshahi, Dinajpur, Nilphamari, Rangpur, Thakurgaon, Sunamganj, Sylhet)	2nd, 5th	DHS	2014	4,494	National and by divisions (Barisal, Chittagong, Dhaka, Khulna, Rajshahi, Rangpur, Sylhet)	3rd, 5th
Bangladesh	WV (ENRICH)	2016	1,323	Districts of Thakurgaon and Panchagarh	2nd	DHS	2014	550	Division of Rangpur	3rd
Bolivia	PLAN	2019	214	Regions of Chuquisaca, La Paz, Cochabamba, and Potosí	4th	DHS	2008	867	Regions of Chuquisaca, La Paz, Cochabamba, and Potosí	4th
Burkina Faso	WUSC	2016	1,005	Regions North, Central-West and East	4th	DHS	2010	2,709	Regions North, Central-West and East	4th
Ethiopia	A&T	2010	3,000	Regions of Tigray and SNNP	4th	DHS	2005	1,800	Regions of Tigray and SNNP	4th
Ethiopia	PLAN (BORN)	2017	905	Zones of North Gondar, South Gondar and West Gojjam and region of Amhara	3rd, 4th	DHS	2016	369	Region of Amhara	4th
Ethiopia	CARE	2016	1,261	Zones of East and West Hararghe and region of Afar	3rd, 4th	DHS	2016	1,630	Regions of Oromia and Afar	4th
Ethiopia	NIMS	2017	440	Regions of Amhara, Tigray, Oromia, Benishangul-Gumuz, and SNNP	4th	DHS	2016	508	Regions of Amhara, Tigray, Oromia, Benishangul-Gumuz, and SNNP	4th
Ethiopia	PLAN	2018	537	Regions of Amhara and SNNP	4th	DHS	2016	1,651	Regions of Amhara and SNNP	4th
Ghana	PLAN (SHOW)	2014	831	Intervention/control districts in the regions of Eastern, Northern, and Volta	2nd	DHS	2014	775	Regions of Eastern, Northern, and Volta	4th
Haiti	PLAN (SHOW)	2016	860	Communes of Fort-Liberté, Ouanaminte, and Trou-du-Nord	2nd	DHS	2012	237	Department of North-east	3rd
Honduras	Red Cross	2007	300	Departments of Copán and Santa Bárbara	3rd	DHS	2005/06	524	Departments of Copán and Santa Bárbara	3rd
India	Eficor	2012	300	District of Pakur	2nd	DHS	2005/06	620	State of Jharkand	3rd
India	IntraHealth	2010	14,090	District of Pakur and Uttar Pradesh	2nd	DHS	2005/06	1,649	States of Jharkand and Uttar Pradesh	3rd
Kenya	NIMS	2017	3,941	Provinces of Rift Valley, Western, Nyanza, Eastern, Coast	3rd	DHS	2014	12,011	Provinces of Rift Valley, Western, Nyanza, Eastern, Coast	3rd
Kenya	Red Cross	2012	154	Districts of East Pokot, Central Pokot, and East Marakwet	2nd	DHS	2008/09	694	Province of Rift Valley	3rd
Kenya	WV (ENRICH)	2016	1,274	Counties of Elgeyo Marakwet and Baringo (subdivision of the before called Rift Valley province)	2nd	DHS	2014	4,760	Province of Rift Valley	3rd
Laos	NIOPH	2018	115	Province of Vientiane	3rd	MICS	2016/17	3,560	Region North	4th
Laos	The World Bank	2016	7,355	Provinces of Phongsaly, Oudomxay, Houaphan, Xaiyabouly, Borlikhamxay	3rd	MICS	2016.5	7,131	Region North	4th
Liberia	Red Cross	2012	783	Counties of Bomi, Gbarpolu, and Grand Gedeh	3rd	DHS	2013	848	Counties of Bomi, Gbarpolu, and Grand Gedeh	3rd
Malawi	CARE	2017	708	Traditional authorities of Kasakula, Kalumo, Dzoole, Kayembe and districts of Ntchisi and Dowa	1st, 3rd	DHS	2015/16	925	Districts of Ntchisi and Dowa	3rd
Mali	PLAN (BORN)	2017	907	Region of Sikasso	4th	DHS	2012/13	714	Region of Sikasso	4th
Mozambique	CARE	2017	1,262	Districts of Funhalouro and Homoine and province of Inhambane	2nd, 3rd	DHS	2011	570	Province of Inhambane	3rd
Mozambique	PLAN	2019	5,921	Districts of Moma, Mogovolas, Nampula, Eráti, Memba, and Nacala Porto	2nd	DHS	2011	358	Province of Nampula	3rd
Myanmar	WV	2016	831	Village of Thabaung	1st	DHS	2015/16	275	Region of Ayeyarwaddy	4th
Nigeria	PLAN (BORN)	2016	1,658	Local Government Areas of Bauchi, Dass, Katagum, Misau, Ningi, Alkaleri, Bogoro, Ganjuwa, Giade, Shira and state of Bauchi	2nd, 3rd	DHS	2013	577	State of Bauchi	3rd
Nigeria	NIMS	2018/19	510	States of Kebbi and Sokoto	3rd	DHS	2018	1,525	States of Kebbi and Sokoto	3rd
Nigeria	PLAN (SHOW)	2016	1,770	Intervention and control districts in the states of Sokoto and Zamfara	2nd	DHS	2013	1,096	States of Sokoto and Zamfara	3rd
Pakistan	NIMS	2017	1,620	Cities of Lodhran, Rajanpur, Jamshoro and Swabi	2nd, 3rd	DHS	2012.5	2,636	Provinces of Punjab, Sindh, and Khyber Pakhtunkhwa	3rd
Pakistan	Red Cross	2012	1,166	Districts of Battagram and Swat and province of Khyber Pakhtunkhwa	2nd, 3rd	DHS	2012/13	1,532	Province of Khyber Pakhtunkhwa	3rd
Pakistan	WV	2017	942	District of Sukkur	2nd	DHS	2012/13	1,591	Province of Sukkur	3rd
Philippines	NIMS	2018	1,418	Provinces of Camarines Norte, Masbate, Antique, Iloilo, Cebu, Bohol, and Zamboanga del Norte	3rd	DHS	2017	352	Provinces of Camarines Norte, Masbate, Antique, Iloilo, Cebu, Bohol, and Zamboanga del Norte	3rd
Senegal	PLAN (SHOW)	2016	828	Intervention/control districts in the regions of Dakar, Ziguinchor, Tambacounda, Kaolack, Louga, Kedougou and Sedhiou	2nd	DHS	2010/11	2,307	Regions of Dakar, Ziguinchor, Tambacounda, Kaolack, Louga, Kedougou and Sedhiou	4th
South Sudan	CMMB	2015	500	County of Nzarai	1st	MICS	2010	770	State of Western Equatoria	3rd
Tanzania	NIMS	2017	215	Regions of Mwanza and Simiyu	4th	DHS	2015/16	408	Regions of Mwanza and Simiyu	4th
Tanzania	PLAN	2017	3,207	Region of Mbeya, and districts of Sumbawanga DC, Sumbawanga MC, Nkasi DC, and Kalambo DC (in the region of Rukwa)	2nd, 4th	DHS	2015/16	282	Regions of Mbeya and Rukwa	4th
Tanzania	WV	2017	1,476	Region of Kigoma	4th	DHS	2015/16	245	Region of Kigoma	4th
Tanzania	WV (ENRICH)	2016	1,399	Districts of Itigi, Manyoni, Ikungi, Kahma, Shinyanga, Kishapu and Ushetu	2nd	DHS	2015/16	556	Regions of Shinyanga and Singida	4th
Vietnam	A&T	2011	4,029	Regions of North Central and Central Coastal area, Northern Midlands - Mountainous area, Central Highlands, Mekong River Delta	4th	MICS	2010/11	7,140	Regions of North Central and Central Coastal area, Northern Midlands - Mountainous area, Central Highlands, and Mekong River Delta	4th
Vietnam	CARE	2015	594	Districts of Bao Lac, Tu Mo Rong, Que Phong and provinces of Nghe An, Cao Bang, and Kon Tum	2nd, 3rd	MICS	2013/14	4,095	Regions of North Central and Central Coastal area, Northern Midlands - Mountainous area, and Central Highlands	4th
Vietnam	Oxfam	2014	1,982	Districts of Da Bac, Hoa Binh, Binh Gia, Lang Son, Phu Cu, and Hung Yen, and provinces of Hoa Binh, Hung Yen, and Lang Son	2nd, 3rd	MICS	2013/14	573	Regions of Northern Midlands - Mountainous area, and Red River Delta	4th
Zambia	CARE	2016	735	Towns of Mpika and Shiwang'andu	2nd	DHS	2013/14	854	Province of Muchinga	3rd

* 1st level represents village, town, locality or traditional authority; 2nd level: district or equivalent; 3rd level: province, state or equivalent; 4th level: region; 5th level: country. DHS: Demographic and Health Surveys; MICS: Multiple Indicator Cluster Surveys; NGO: non-governmental organization.

We also retained the location of data collection (e.g. country, region, province, district, or/and village) and geographical level. These geographical levels of data aggregation were defined as: (1) the smallest geographical subdivision in a country (village, town, locality, traditional authority); (2) district or district council (larger than a village but smaller than the third level); (3) province, state, department, county or district (if it refers to a division equivalent to province or state); (4) region (combining several units of level 3); (5) country level.

### Data from DHS and MICS surveys

We matched 25 DHS and 3 MICS surveys (from Vietnam, Laos, and South Sudan) with 46 NGO baseline reports ( Table 1). We used the most recent DHS/MICS survey carried out prior to the NGO baseline survey, with some surveys matching more than one NGO survey.

Indicators from DHS/MICS were calculated following the methods recommended by DHS/MICS accounting for weighting and sample selection ( Croft *et al.*, 2018). Wherever possible, we used the methods employed by the NGO to create the matching DHS/MICS indicator. For instance, if the NGO baseline survey included women of reproductive age and their children aged 0-24 months living in the district of Homoine in Mozambique, we extracted the same sample from the DHS/MICS. In the absence of representative data from the same geographical level, we used DHS/MICS data from the next level up in the geopolitical hierarchy to match the lower level from the NGO. For instance, if data from the district of Homoine were not available in the DHS, we used data from the province of Inhambane (one level up).

### Indicators retained for analysis

We matched similar indicators from NGO baseline reports with DHS/MICS wherever available and excluded those that had no match in the DHS/MICS datasets. Table 2 provides an example of how the data were matched for the indicator “Woman received at least three antenatal care visits (ANC) during last pregnancy”.

**Table 2.** Example of how the estimates from NGO and DHS/MICS were matched for the indicator “Woman had at least three ANC visits during last pregnancy (%)”.

				NGO					DHS/MICS				
Country	Region	Province	District	Source	Year	Level	n	Estimate	Source	Year	Level	n	Estimate
Ethiopia	Amhara+Tigray+ Oromia+Benishangul-Gumuz+SNNP	-	-	NIMS	2017.5	4th	409	77.0	DHS	2016	4th	4017	50.0
India	-	Jharkhand	Pakur	Eficor	2012	2nd	300	29.3	DHS	2005.5	3rd	618	35.9
India	-	Jharkhand	Jharkhand	IntraHealth	2010	2nd	5203	47.0	DHS	2005.5	3rd	320	36.6
India	-	Uttar Pradesh	Uttar Pradesh	IntraHealth	2010	2nd	8860	50.0	DHS	2005.5	3rd	1307	25.6
Pakistan	-	Khyber Pakhtunkhwa	Battagram	Red Cross	2012	2nd	583	22.4	DHS	2012.5	3rd	1529	37.3
Pakistan	-	Khyber Pakhtunkhwa	Swat	Red Cross	2012	2nd	583	36.3	DHS	2012.5	3rd	1529	37.3
Tanzania	Kigoma	-	-	World Vision	2017	4th	485	67.7	DHS	2015.5	4th	278	69.6
Vietnam	North Central and Central Coastal area	Nghe An	Que Phong	CARE	2015	2nd	196	77.6	MICS	2013.5	4th	300	92.8
Vietnam	Northern Midlands - Mountainous area	Cao Bang	Bao Lac	CARE	2015	2nd	198	72.2	MICS	2013.5	4th	230	72.2
Vietnam	Central Highlands	Kon Tum	Tu Mo Rong	CARE	2015	2nd	200	71.0	MICS	2013.5	4th	109	68.5
Vietnam	North Central and Central Coastal area, Northern Midlands - Mountainous area, Central Highlands	Nghe An+Cao Bang+Kon Tum	-	CARE	2015	3rd	594	73.6	MICS	2013.5	4th	640	81.2
Vietnam	Northern Midlands - Mountainous area	Hoa Binh	Da Bac+Hoa Binh	Oxfam	2014	2nd	472	94.7	MICS	2013.5	4th	230	72.2
Vietnam	Red River Delta	Hung Yen	Phu Cu+Hung Yen	Oxfam	2014	2nd	743	98.6	MICS	2013.5	4th	343	92.6
Vietnam	Northern Midlands - Mountainous area	Lang Son	Binh Gia+Lang Son	Oxfam	2014	2nd	767	93.9	MICS	2013.5	4th	230	72.2
Vietnam	Northern Midlands - Mountainous area, Red River Delta	Hoa Binh+Hung Yen+Lang Son	-	Oxfam	2014	3rd	1982	95.9	MICS	2013.5	4th	573	84.4

* 1st level represents village, town, locality or traditional authority; 2nd level: district or equivalent; 3rd level: province, state or equivalent; 4th level: region; 5th level: country. ANC: antenatal care; DHS: Demographic and Health Surveys; MICS: Multiple Indicator Cluster Surveys; NGO: non-governmental organization.

In total there were 129 indicators ( Table 3) from eight main groups including child anthropometry, child diet, child health, household characteristics, household wealth, maternal characteristics, maternal health, and WASH. We excluded estimates based on fewer than ten observations (n=64), in either the DHS/MICS or NGO data, retaining a total of 1,996 pairs of NGO-DHS/MICS indicators for analyses.

**Table 3.** List of indicators collected by group and subgroup *

Group	Subgroup	N indicators in subgroup	Details
Child anthropometry	Stunting	19	There are separate indicators by age groups, and for boys and girls (separated and combined)
Child anthropometry	Underweight	22	There are separate indicators by age groups, and for boys and girls (separated and combined)
Child diet	Ate 4+ food groups	5	By age group and by breastfeeding status, and combined
Child diet	Bottle fed yesterday	3	By age group, and combined
Child diet	Consumed iron-rich foods	4	By age group, and combined
Child diet	Consumed vitamin A-rich foods	1	
Child diet	Continued breastfeeding	4	By age group
Child diet	Exclusive breastfeeding: 0-6 m	3	Boys and girls separately and combined
Child diet	Initiation of breastfeeding within 1 hour of birth	3	Boys and girls separately and combined
Child diet	Receiving solid, semi-solid or soft foods: 6-8 m	1	
Child health	Child took supplement/vaccine	4	Child received iron or vitamin A supplements, child received DPT and measles by 12 months of age, newborn protected by tetanus vaccine
Child health	Diarrhea in last two weeks	6	By age group (diarrhea in 0-5m is separate subgroup)
Child health	Diarrhea in the last two weeks: 0-5 m	1	
Child health	Received diarrhea treatment	4	Those with diarrhea received ORS, ORT, homemade fluids, ORS+ zinc
Child health	For those with diarrhea in last 2 weeks, given more to drink	1	
Child health	For those with diarrhea in last 2 weeks, given more to eat	1	
HH characteristics	Individuals who have ever been married	1	
HH characteristics	Head of household is male	1	
HH characteristics	Household has electricity	1	
HH characteristics	Urban residence	1	
HH wealth	Household has a car		
HH wealth	Household has agricultural land/bike/phone	3	Household has land, bike, phone
HH wealth	Household has animals	6	Household has cattle, chickens, goats, horses, livestock, poultry, sheep
Maternal characteristics	Woman able to read	1	
Maternal characteristics	Woman never attended school	1	
Maternal health	Birth at a health facility/assisted by a skilled birth attendant (SBA)	3	Last birth at health facility, attended by SBA, assisted by SBA
Maternal health	Woman consumed/received iron supplements	5	Woman received iron supplements, woman consumed iron supplements on 1+, 90+, 100+, 150+ days
Maternal health	Woman received antenatal care (ANC)	4	In last pregnancy, woman had ANC in first trimester, woman had 1+, 3+, 4+ ANC visits
Maternal health	Woman received postnatal care (PNC)	3	Woman received PNC, Woman received PNC with 2 days/3 days of birth
Maternal health	Woman's antenatal care (ANC) content	5	During ANC woman had blood/urine test, blood pressure taken, received 2+ TT vaccines, was weighed.
WASH	Handwash station has ash/sand/soap/water	3	Household handwash station has ash/sand, water, soap
WASH	Household dispose child stool in toilet/latrine	1	
WASH	Household has improved drinking water	1	
WASH	Household has improved sanitation	1	
WASH	Household shares toilet	1	
WASH	Household treats drinking water	2	Household bleaches/boils drinking water
WASH	30+ min for household to obtain drinking water	1	

* for a complete list of all the indicators see Table 2 in HealthBridge (2020). HH: household; WASH: Water, Sanitation, and Hygiene; DPT: diphtheria, pertussis and tetanus; ORS: oral rehydration salts; ORT: oral rehydration therapy; SBA: skilled birth attendant; ANC: antenatal care; PNC: postnatal care; TT: tetanus toxoid.

After collating the data, we grouped similar indicators into 37 subgroups ( Table 3) on the basis of whether they had similar definitions/concepts (e.g. stunting prevalence in different age groups). We refined the grouping by using scatterplots of the difference of estimates by year difference and geographical level difference to check if any indicators differed widely from others in the grouping. After assessing the indicators graphically, we separated “Diarrhea in the last two weeks: 0-5m” from the same indicator for other age groups since the differences of estimates were closer to zero for this age group than the others. We also separated “Household has a car” from the subgroup “Household has agricultural land/bike/phone” since car ownership was much lower than ownership of other assets.

### Analysis

*NGO versus DHS/MICS*

We subtracted NGO from DHS/MICS estimates to calculate difference and absolute difference between estimates.

To compare data from NGO and DHS/MICS we used: same or different season of data collection; number of years difference between data collection (DHS/MICS year - NGO year); and number of geographical levels difference (DHS/MICS level - NGO level). If data collection spanned two years, for instance data collection started in 2013 and was completed in 2014, the year of data collection was coded as “2013.5”. Geographical level difference was calculated by subtracting the NGO level from DHS/MICS level. For example, we subtracted district level data available from the Mozambique NGO survey (level=2) from province level data collected in the DHS (level=3), making the geographical level difference one. We grouped geographical level differences as: no difference; one level difference; 2-3 levels difference.

We plotted how difference and absolute difference between DHS/MICS and NGO estimates varied with the indicator and indicator grouping. We used Analysis of Variance (ANOVA) to partition the variance of difference or absolute difference between estimates (DHS/MICS estimate - NGO estimate) by indicator, geographical level difference (as 0,1,2+), year difference (continuous), and season (same season, different season, season unknown).

*DHS versus DHS*

In order to better understand the contribution of difference in methods employed in the different sources of survey data (DHS/MICS and NGO) to the resulting difference in estimates, we repeated the analyses used to compare DHS/MICS and NGO estimates but this time comparing DHS data from one country, year and geographical level to a different year and/or geographical level from the same country. The assumption is that the DHS methods are similar between years and geographical levels, whereas DHS/MICS and NGOs may use somewhat different methods. There is a level of discordance between DHS/MICS and NGO estimates, and there would also be discordance between two DHS estimates. The difference between DHS/MICS-NGO discordance and DHS-DHS discordance will not be due to difference in years, or geographical levels, but rather due to difference in methods.

For the DHS-DHS comparisons, we compiled DHS data from the seven countries that contributed the most pairs in the DHS/MICS-NGO dataset: Bangladesh, Ethiopia, Kenya, Malawi, Pakistan, Tanzania, and Zambia. Retaining the same indicators as in the DHS/MICS - NGO comparisons, we calculated estimates for different geographical levels, i.e. at the country level, and for each region, province and district available. For this analysis, we included district data to mimic the NGO data, even though these estimates are not always representative at this level in the DHS. We excluded indicators based on a sample size smaller than ten observations (n=26,539).

We matched DHS indicators from different cycles and geographical levels using different combinations mimicking the actual DHS/MICS-NGO scenarios: indicators from the same level but different years (Scenario 1), indicators from the same year but different levels (Scenario 2), and indicators from different years and levels (Scenario 3). To mimic the NGO data, we used data from the most recent cycle and the lower geographical levels, whereas to represent the comparative DHS data we used older DHS cycle and higher geographical level data. Using DHS data only, we were not able to simulate a scenario where DHS/MICS and NGO data were from the same year and geographical level. Table 4 provides an example of how we compared the estimates for an ANC indicator in Zambia using 31 pairs from DHS in the three scenarios for this one country. Repeating across all indicators and all countries yielded 109,251 pairs of DHS-DHS indicators.

**Table 4.** Example of how the estimates for the indicator “Woman had at least three ANC visits during last pregnancy (%)” in Zambia were compared using DHS data from different/same years and geographical levels.

		Data from DHS earlier year or higher level				Data from DHS later year or lower level			
	Province	Level	Year	n	Estimate	Level	Year	n	Estimate
**DHS data from different years but same geographical level** **(Scenario 1)**	Central	3rd	2013.5	789	89.2	3rd	2018	746	89.0
	Copperbelt	3rd	2013.5	853	91.3	3rd	2018	730	91.4
	Eastern	3rd	2013.5	1,136	89.1	3rd	2018	875	93.6
	Luapula	3rd	2013.5	988	88.2	3rd	2018	817	91.9
	Lusaka	3rd	2013.5	904	88.5	3rd	2018	818	89.3
	Muchinga	3rd	2013.5	850	86.7	3rd	2018	661	91.2
	North Western	3rd	2013.5	927	86.1	3rd	2018	608	92.6
	Northern	3rd	2013.5	981	85.4	3rd	2018	692	90.6
	Southern	3rd	2013.5	1,036	89.9	3rd	2018	746	94.5
	Western	3rd	2013.5	793	85.2	3rd	2018	612	90.4
	All	5th	2013.5	9,257	88.5	5th	2018	7,305	91.5
**DHS data from the same year but different geographical levels** **(Scenario 2)**	Central	5th	2013.5	9,257	88.5	3rd	2013.5	789	89.2
	Copperbelt	5th	2013.5	9,257	88.5	3rd	2013.5	853	91.3
	Eastern	5th	2013.5	9,257	88.5	3rd	2013.5	1,136	89.1
	Luapula	5th	2013.5	9,257	88.5	3rd	2013.5	988	88.2
	Lusaka	5th	2013.5	9,257	88.5	3rd	2013.5	904	88.5
	Muchinga	5th	2013.5	9,257	88.5	3rd	2013.5	850	86.7
	North Western	5th	2013.5	9,257	88.5	3rd	2013.5	927	86.1
	Northern	5th	2013.5	9,257	88.5	3rd	2013.5	981	85.4
	Southern	5th	2013.5	9,257	88.5	3rd	2013.5	1,036	89.9
	Western	5th	2013.5	9,257	88.5	3rd	2013.5	793	85.2
**DHS data from different years and different geographical levels** **(Scenario 3)**	Central	5th	2013.5	9,257	88.5	3rd	2018	746	89.0
	Copperbelt	5th	2013.5	9,257	88.5	3rd	2018	730	91.4
	Eastern	5th	2013.5	9,257	88.5	3rd	2018	875	93.6
	Luapula	5th	2013.5	9,257	88.5	3rd	2018	817	91.9
	Lusaka	5th	2013.5	9,257	88.5	3rd	2018	818	89.3
	Muchinga	5th	2013.5	9,257	88.5	3rd	2018	661	91.2
	North Western	5th	2013.5	9,257	88.5	3rd	2018	608	92.6
	Northern	5th	2013.5	9,257	88.5	3rd	2018	692	90.6
	Southern	5th	2013.5	9,257	88.5	3rd	2018	746	94.5
	Western	5th	2013.5	9,257	88.5	3rd	2018	612	90.4

* 3rd level represents province level data and 5th level represents country-level data.

ANC: antenatal care; DHS: Demographic and Health Surveys.

We calculated the difference and absolute difference between these pairs of estimates, mimicking the scenarios from the DHS/MICS-NGO data. Table 5 summarises the DHS cycles included as well as the geographical level comparison for each scenario in each of the seven countries.

**Table 5.** Demographic and Health Survey (DHS) cycles and geographical level comparison included in the DHS vs DHS analysis.

	Scenario 1 (N=9,024)		Scenario 2 (N=56,185)		Scenario 3 (N=44,042)	
Country	DHS cycle	Geographical level comparison	DHS cycle	Geographical level comparison	DHS cycle	Geographical level comparison
Bangladesh	2011 2014	3rd-3rd 5th-5th	2014	3rd-2nd 5th-2nd 5th-3rd	2011 2014	3rd-2nd 5th-2nd 5th-3rd
Ethiopia	2011 2016	3rd-3rd 5th-5th	2016	5th-3rd	2011 2016	5th-3rd
Kenya	2008.5 2014	3rd-3rd 5th-5th	2014	5th-3rd	2008.5 2014	5th-3rd
Malawi	2010 2015.5	3rd-3rd 4th-4th 5th-5th	2015.5	4th-3rd 5th-3rd 5th-4th	2010 2015.5	4th-3rd 5th-3rd 5th-4th
Pakistan	2006.5 2012.5	3rd-3rd 5th-5th	2012.5	3rd-2nd 5th-2nd 5th-3rd	2006.5 2012.5	3rd-2nd 5th-2nd 5th-3rd
Tanzania	2010 2015.5	4th-4th 5th-5th	2015.5	4th-2nd 5th-2nd 5th-4th	2010 2015.5	4th-2nd 5th-2nd 5th-4th
Zambia	2013.5 2018	3rd-3rd 5th-5th	2013.5	5th-3rd	2013.5 2018	5th-3rd

Geographical level comparison: geographical level from older cycle vs geographical level from most recent cycle included.

2nd level represents district or equivalent; 3rd level: province, state or equivalent; 4th level: region; 5th level: country.

Scenario 1: DHS data from different years compared using the same geographical levels.

Scenario 2: DHS data from the same year compared using different geographical levels.

Scenario 3: DHS data from different years compared using different geographical levels.

Finally, as with DHS/MICS vs NGO estimates, we used ANOVA to partition the variance of difference or absolute difference between DHS estimates by indicator, geographical level difference, and year difference. We did not include season in this analysis since most DHS data are collected during the same season within a country.

### Simulations

We simulated a situation where the only source of imprecision of the indicator’s measures would be from sampling error, in order to separate this known and estimable source of error from other sources of error that lead to differences in indicator estimates. The simulation samples from a "true" prevalence (p) of 1%, 10%, 20%, 30%, 40%, 50%, 60%, 70%, 80%, 90%, and 99%. We assumed an n of 500, which was a typical sample size of both DHS and NGO samples in our data set. We then generated a “Baseline Estimate 1” (to mimic the DHS/MICS estimates) by drawing randomly from a binomial distribution with mean n*p and variance np(1-p). A “Baseline estimate 2” (to mimic the NGO estimate) was generated in the same way, and the difference between the first and second estimate was calculated. We ran 1,000 iterations to estimate the distribution of the differences.

In order to investigate how absolute differences vary by the nature of the point prevalence estimates we used box plots to compare simulated, DHS-DHS and DHS/MICS-NGO absolute differences.

All data were compiled in Microsoft Excel 15 and analyzed with SAS 9.4.

This study respects current research ethics standards and it was approved by the Health Research Ethics Board of the Université de Montréal (CERSES-19-030-D).

## Results

The NGO reports often presented over 100 indicators in their baseline reports. On average, 18 of their indicators were also available in the DHS/MICS datasets. The estimate sample size for the NGO surveys ranged from 12 to 16,530 and from 10 to 98,446 for the DHS/MICS. Table 6 presents, by indicator subgroup, mean DHS/MICS and NGO percentage prevalence estimates, mean difference between pairs (DHS/MICS minus NGO) and percentage of differences falling within 5 and 20 percentage points. Some subgroups have mean difference close to zero, but almost all have at least some pairs that are widely different (not within 20%). Fifteen subgroups had positive (DHS<NGO) and 21 had negative (DHS>NGO) mean differences, but we identified no meaningful pattern in which indicators were negative and which were positive, and all the differences (except for consumption of vitamin A-rich foods) were within 1 standard deviation of 0.

**Table 6.** DHS/MICS and NGO estimates, difference between estimates (DHS/MICS minus NGO) and proportion of estimates within 5% and 20% difference by subgroup of indicators.

		DHS/MICS estimate		NGO estimate		Difference between estimates				Percentage of indicator pairs with difference within:	
Subgroup	N	Mean	SD	Mean	SD	Mean	SD	Min	Max	5%	20%
**Child anthropometry**											
Stunting (%)	131	30.6	10.4	36.4	11.9	-5.7	9.8	-42.1	20.6	38.2	93.1
Underweight (%)	131	26.9	12.8	18.5	8.7	8.5	11.1	-15.2	32.3	30.5	80.9
**Child diet**											
Ate 4+ food groups (%)	67	21.3	9.2	22.6	12.3	-1.3	12.3	-23.2	28.9	25.4	94.0
Bottle fed yesterday (%)	33	8.8	6.4	6.9	9.4	1.9	6.8	-20.8	13.1	63.6	97.0
Consumption of iron-rich foods (%)	30	28.0	12.2	18.6	15.5	9.4	19.0	-39.2	52.3	10.0	70.0
Consumption of vit A-rich foods (%)	4	30.8	25.1	19.1	24.5	11.7	3.6	7.7	16.1	0.0	100.0
Continued breastfeeding (%)	32	82.6	16.8	79.0	22.2	3.6	10.3	-10.8	32.4	53.1	90.6
Exclusive breastfeeding: 0-6 m (%)	60	42.0	17.4	62.1	20.0	-20.1	20.2	-60.1	22.2	16.7	46.7
Initiation of breastfeeding within 1 hour of birth (%)	64	67.6	17.0	59.0	18.4	8.6	18.7	-33.5	55.2	35.9	75.0
Receiving foods: 6-8 m (%)	18	69.8	18.2	66.1	23.2	3.7	30.3	-53.4	50.6	16.7	44.4
**Child health**											
Child received supplement/vaccine (%)	10	57.6	21.7	65.7	25.8	-8.1	15.7	-37.9	14.6	20.0	80.0
Diarrhea in the last two weeks (%)	86	19.1	9.4	30.7	20.2	-11.6	14.5	-46.8	15.8	33.7	70.9
Diarrhea in the last two weeks: 0-5 m (%)	11	9.8	7.0	14.9	7.8	-5.1	3.7	-10.4	2.8	54.5	100.0
Diarrhea treatment (%)	31	36.0	24.3	41.7	17.8	-5.6	22.9	-50.5	55.5	19.4	61.3
Diarrhea, given more to drink (%)	22	19.3	10.7	27.0	16.8	-7.7	21.4	-52.3	30.1	13.6	63.6
Diarrhea, given more to eat (%)	14	8.8	4.4	6.8	4.9	2.0	3.9	-6.0	7.9	64.3	100.0
**Household characteristics**											
Ever married (%)	57	96.5	9.6	85.8	14.5	10.8	10.3	-5.0	31.7	42.1	77.2
Household has electricity (%)	20	43.8	40.6	44.6	38.8	-0.8	9.2	-21.9	15.4	60.0	95.0
Head of household is male (%)	78	85.6	11.6	87.9	8.4	-2.3	8.8	-25.3	25.8	56.4	92.3
Urban residence (%)	12	23.0	11.9	31.8	11.8	-8.8	15.5	-36.3	12.2	33.3	75.0
**Household wealth**											
Household has a car (%)	46	2.0	2.4	1.8	3.9	0.2	2.7	-12.4	5.3	91.3	100.0
Household has agricultural land/bike/phone (%)	150	56.1	28.4	50.5	29.5	5.7	14.4	-52.9	41.7	34.7	82.7
Household has animals (%)	73	41.9	25.3	37.9	23.2	4.0	9.5	-24.0	29.5	41.1	93.2
**Maternal characteristics/health**											
Woman able to read (%)	8	33.2	22.9	27.3	14.6	5.9	11.4	-6.4	21.0	12.5	87.5
Woman never attended school (%)	58	40.3	29.9	36.5	28.5	3.8	12.5	-43.7	53.1	37.9	94.8
Birth at a health facility/assisted by skilled birth attendant (%)	127	46.5	22.7	59.1	24.0	-12.6	15.6	-45.0	49.0	17.3	69.3
Woman received/consumed iron supplements (%)	63	49.7	28.4	49.8	32.8	-0.2	17.8	-38.0	55.5	33.3	76.2
Woman received antenatal care (%)	162	58.2	24.5	63.2	23.7	-5.0	16.1	-35.7	54.6	24.1	75.3
Woman received postnatal care (%)	51	41.6	17.1	44.0	23.6	-2.4	29.2	-65.6	78.0	5.9	51.0
Woman’s ANC content (%)	56	55.3	21.4	57.2	26.2	-1.9	27.6	-60.9	46.4	14.3	48.2
**WASH**											
Household dispose child stool in toilet/latrine (%)	14	55.9	27.5	65.4	26.8	-9.5	14.8	-38.7	20.3	28.6	78.6
Household has improved drinking water (%)	87	64.5	26.8	70.3	24.2	-5.7	17.2	-54.6	51.9	34.5	81.6
Household has improved sanitation (%)	82	33.5	21.4	40.8	27.8	-7.3	26.8	-62.4	77.5	12.2	53.7
Household shares toilet (%)	11	31.7	13.9	28.5	13.7	3.2	18.0	-21.2	47.7	27.3	81.8
Household treats drinking water (%)	52	8.4	8.7	15.2	14.1	-6.8	15.4	-50.8	19.1	53.8	86.5
Handwash station has ash/sand/soap/water (%)	25	20.8	12.5	30.5	21.5	-9.8	16.8	-57.8	26.6	24.0	76.0
Time to obtain drinking water 30+ min (%)	20	33.4	12.1	36.1	20.2	-2.7	22.8	-46.2	58.3	25.0	75.0

DHS: Demographic and Health Surveys; MICS: Multiple Indicator Cluster Surveys; NGO: non-governmental organization; SD: standard deviation; ANC: antenatal care; WASH: Water, Sanitation and Hygiene.

**Figure 1.**
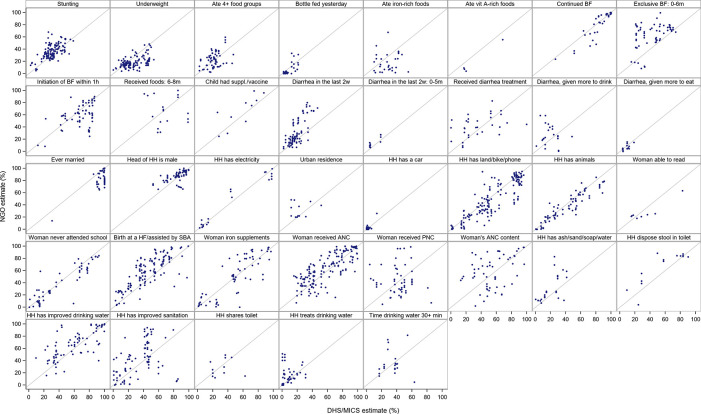
DHS/MICS estimate by NGO estimate by subgroup of indicators. Abbreviations: BF: breastfeeding; HH: household; HF: health facility; SBA: skilled birth attendant; ANC: antenatal care; PNC: postnatal care; DHS: Demographic and Health Surveys; MICS: Multiple Indicator Cluster Surveys; NGO: non-governmental organization.

**Figure 2.**
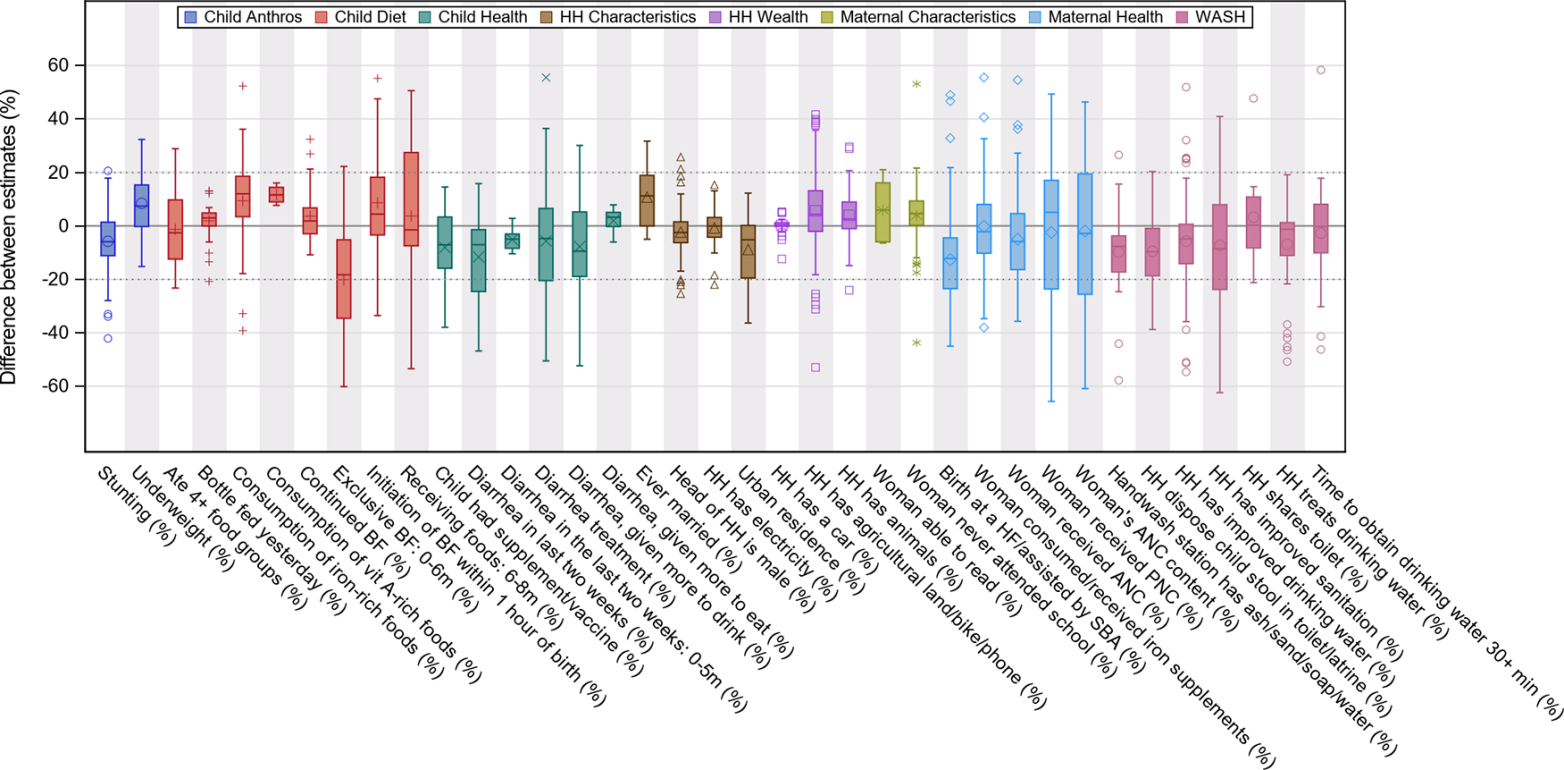
Difference between estimates (DHS/MICS minus NGO) by subgroup of indicators. Abbreviations: Anthros: anthropometry indicators; HH: household; WASH: Water, Sanitation, and Hygiene; BF: breastfeeding; HF: health facility; SBA: skilled birth attendant; ANC: antenatal care; PNC: postnatal care; DHS: Demographic and Health Surveys; MICS: Multiple Indicator Cluster Surveys; NGO: non-governmental organization.

Figure 1 presents the scatterplots of NGO against DHS/MICS estimates by subgroup of indicators. For all subgroups, there was some correlation between the DHS/MICS and NGO estimates. Figure 2 shows the boxplot distribution of the mean difference between estimates by subgroup. The only subgroups that had *all* the pairs of indicators within ±20% were “Consumption of vitamin A-rich foods”, “Bottle fed yesterday”, “Diarrhea in the last two weeks: 0-5m”, “Diarrhea in the past two weeks: given more to eat”, and “Household has a car”. Other indicators that had *most* of their pairs within ±20% were “Household treats drinking water” and “Ever married”. All the indicators with the smallest differences between estimates had very low or very high prevalence ( Table 6), except for “Consumption of vitamin A-rich foods” (that was based on only four pairs of estimates).

Table 7 summarizes the absolute differences between DHS/MICS and NGO, and between DHS and DHS. They are summarized according to the similarity of data collection timing (year and season), geographical level, and sample size. Using the absolute difference enabled us to see the size of the difference without taking the direction into account. The absolute difference between DHS/MICS and NGO estimates increases as year difference increases, as geographical levels difference increase, and as sample sizes decrease. The differences between DHS and DHS show similar patterns in terms of broad geographical level, sample size, and ≥3.5 years versus 0 to 3 years’ time differences.

**Table 7.** Absolute difference of estimates by year difference, season, geographical level, and sample size.

	DHS/MICS vs NGO					DHS vs DHS				
Variable	N	Mean	SD	Median	IQR	N	Mean	SD	Median	IQR
**Year difference**										
≤1 year	495	11.6	10.4	9.2	12.6	56185	10.1	10.2	6.9	11.7
1.5-3 years	860	12.8	12.8	8.4	15.9	8024	9.3	9.1	6.6	10.7
≥3.5 years	641	13.8	13.2	10.1	15.1	45042	13.6	13.9	9.2	15.3
**Season**										
Same season	1153	13.1	12.8	9.0	14.4	-	-	-	-	-
Different season	603	11.8	11.2	8.5	14.6	-	-	-	-	-
Season unknown	240	14.2	13.0	10.3	16.2	-	-	-	-	-
**Geographical level difference**										
0	677	12.5	12.6	8.3	14.2	9024	10.1	11.5	6.2	11.4
1	897	13.1	12.3	9.6	15.4	30275	10.5	10.9	7.1	11.9
2+	422	12.8	12.2	9.0	14.9	69952	12.1	12.4	8.2	13.8
**Geographical level 1** ^**a**^^**,**^^**b**^										
Country	14	7.7	7.7	3.9	6.1	61230	11.9	12.2	8.1	13.7
Region	1259	13.1	12.9	9.0	15.9	25248	10.9	12.2	7.0	12.0
Province	723	12.4	11.4	9.3	13.4	22773	10.8	10.8	7.5	12.6
**Geographical level 2** ^**c**^^**,**^^**d**^										
Country	14	7.7	7.7	3.9	6.1	896	7.0	8.7	3.9	7.2
Region	369	12.6	12.2	8.7	14.2	8826	11.3	13.1	7.1	12.7
Province	422	12.5	12.4	9.0	13.7	30875	9.1	9.7	5.9	10.1
District	963	13.0	12.3	9.3	15.2	68654	12.6	12.6	8.8	14.5
Village	228	13.5	13.3	8.6	17.6	-	-	-	-	-
**Sample size 1** ^**a**^^**,**^^**b**^										
Tertile 1 (n ^**a**^=335, n ^**b**^=709)	663	14.1	13.1	9.8	16.6	36418	11.5	12.1	7.8	12.6
Tertile 2	656	12.9	12.4	9.3	15.0	36695	11.2	11.7	7.5	12.9
Tertile 3 (n ^**a**^=772, n ^**b**^=5282)	677	11.6	11.5	8.2	13.3	36138	11.7	12.1	7.8	13.8
**Sample size 2** ^**c**^^**,**^^**d**^										
Tertile 1 (n ^**c**^=236, n ^**d**^=37)	664	14.8	13.7	10.4	17.5	36480	13.7	13.0	10.0	14.9
Tertile 2	668	12.0	12.0	8.1	14.0	36407	11.7	11.9	8.0	13.1
Tertile 3 (n ^**c**^=757, n ^**d**^=104)	664	11.7	11.1	8.7	13.4	36364	9.0	10.4	5.4	10.3

^a^ For the DHS/MICS - NGO comparison, refers to the DHS/MICS data.

^b^ For the DHS - DHS comparison, refers to the DHS data from the higher geographical level and earlier survey year.

^c^ For the DHS/MICS - NGO comparison, refers to the NGO data.

^d^ For the DHS - DHS comparison, refers to the data mimicking the NGO (from the lower geographical level and more recent survey year).

DHS: Demographic and Health Surveys; MICS: Multiple Indicator Cluster Surveys; NGO: non-governmental organization.

Table 8 shows the partition of variation results from DHS/MICS vs NGO and DHS vs DHS comparison. For DHS/MICS vs NGO about 15% of the variance was attributed to the indicator and less than 1% attributed to geographical level, year and season difference. For DHS vs DHS, geographical level and year account for more variation in absolute difference (1.25 and 4.5% respectively). However, in all cases, most (>82%) of the variance was unattributed, that is, it remained unexplained by the model.

**Table 8.** Partition of variance of difference and absolute difference between estimates by indicator, geographical level difference, year difference, and season.

	DHS/MICS vs NGO						DHS vs DHS				
	Percent variance due to (%):						Percent variance due to (%):				
Dependent variable	n	Indicator	Geo. level difference	Year difference	Season	Other	n	Indicator	Geo. level difference	Year difference	Other
Difference	1996	16.69	0.00	0.61	0.02	82.67	109251	6.48	0.04	0.00	93.48
Absolute difference	1996	16.76	0.00	0.23	0.15	82.87	109251	12.61	1.25	4.50	81.63

Results from the ANOVA models.

Model: difference or absolute difference between estimates by indicator, geographical level difference (0,1,2+), year difference (continuous), and season (same season, different season, season unknown - in NGO vs DHS/MICS comparison only).

DHS: Demographic and Health Surveys; MICS: Multiple Indicator Cluster Surveys; NGO: non-governmental organization; ANOVA: Analysis of Variance.

**Figure 3.**
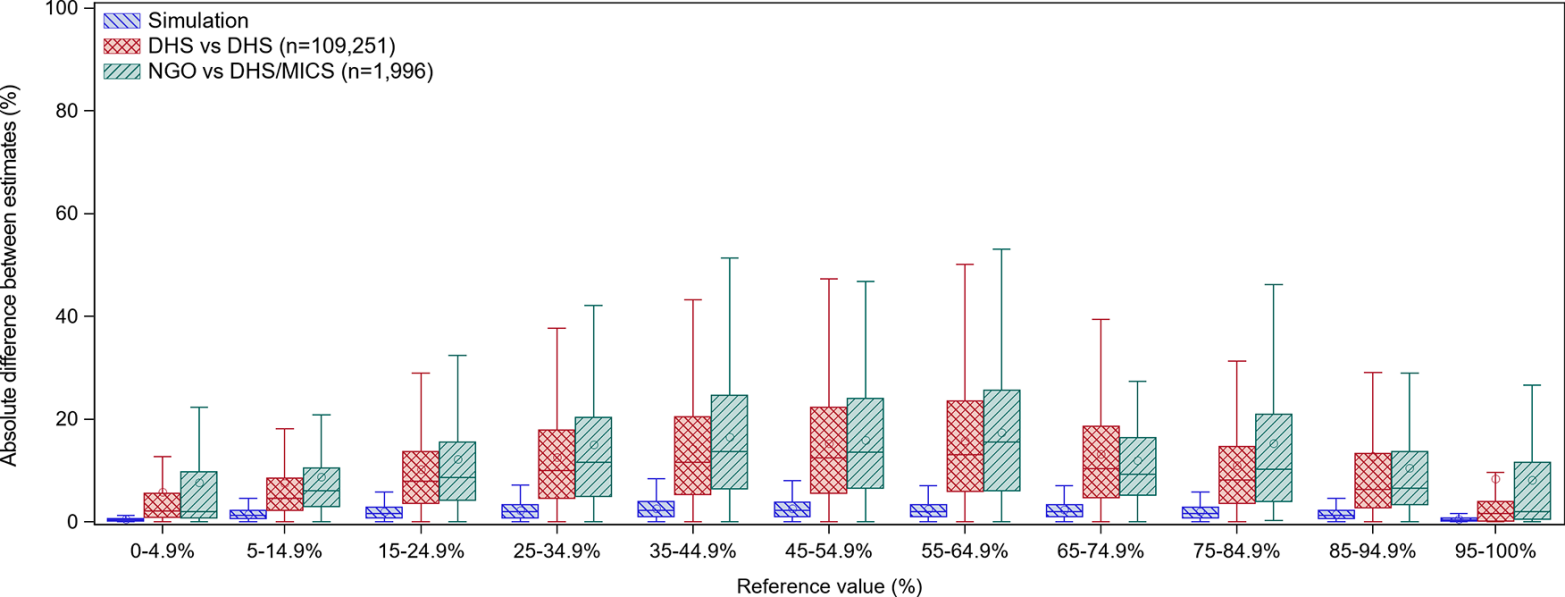
Box plot of absolute difference between NGO and DHS/MICS estimates by the reference value. Absolute difference between estimates calculated as:
Simulation: Simulated estimate 1 - Simulated estimate 2DHS vs DHS: DHS estimate - DHS mimicking the NGO estimate (lower geographical level, more recent year of data collection)DHS/MICS vs NGO: DHS/MICS estimate - NGO estimateReference value: DHS or the estimate mimicking DHS (higher geographical level, earlier year of data collection) Abbreviations: DHS: Demographic and Health Surveys; MICS: Multiple Indicator Cluster Surveys; NGO: non-governmental organization.

Results from all three comparisons, DHS/MICS - NGO, DHS - DHS, and Simulations, are shown in Figure 3 as boxplots of the absolute difference between estimates by the indicator reference value (the DHS estimate or the estimate simulating DHS). The distribution of absolute differences is similar between DHS/MICS - NGO and DHS - DHS, with DHS/MICS - NGO showing only a slightly larger spread. For all three types of comparisons, the distribution of the absolute difference between estimates is narrower in the extremes and larger when the reference value is between 35% and 65%. Since the simulated sampling error differences are small (range <10%), only a small proportion of the differences can be attributed to sampling error.

## Discussion

Our study showed that many indicators presented large differences between NGO and DHS/MICS estimates. Almost all indicators had at least some pairs that were widely different. Only about 33% of the pairs of indicators were within 5%, and about 80% of the pairs of indicators were within 20%. Agreement between indicators was higher when comparing indicators that had low or high prevalence (e.g. <15% or >85%), which is consistent with sampling theory, but throughout the prevalence range, the distribution of differences in the DHS/MICS-NGO and DHS-DHS comparisons is larger than that found from sampling error alone (reflected in the simulation distribution). An NGO could obtain an accurate estimate using DHS/MICS data for indicators with expected values close to 0% or 100%.

We had hoped that if DHS/MICS and NGO estimates were similar, then NGOs could forego baseline data collection and use as a substitute DHS/MICS estimates, or estimates from some other publicly available dataset instead, saving NGO time and money, and reducing respondent burden. While we cannot give a blanket recommendation that DHS and MICS could always replace NGO baseline surveys, there are at least some situations where DHS/MICS could be used to the NGO’s advantage: when the estimate is expected to be less than 15% or above 85%; when the indicator of interest is one of the few with consistent similarity between DHS/MICS and NGO estimates; and when the NGO has tolerance for estimates of low or unknown accuracy.

We had hypothesized that publicly available data can provide estimates of baseline conditions similar to those reported in NGO baseline reports when matched as closely as possible for location, year, and season of data collection. From the descriptive analyses, we found that as year difference increased, the mean difference between estimates slightly increased, and estimates derived from lower geographical levels (such as village or district from NGO and province for DHS/MICS) contributed to a higher mean absolute difference between estimates. In general, larger sample sizes were obtained at higher geographical levels and the larger the sample size (with their smaller sampling error) from DHS/MICS or NGO, the smaller the mean absolute difference between estimates. This meant that the advantage of geographical proximity is offset by the larger sampling error associated with small sample sizes. Whether the seasons of data collection were matched or different did not make a measurable difference to the similarity between estimates.

However, the partition of variance analyses showed that DHS/MICS and NGO estimates differed, for the most part, in unpredictable ways, and geographical levels, years difference and seasons explained only a small part of the variation.

We hypothesize that large differences between estimates from NGO baseline reports and DHS/MICS data are due to three main reasons:

(i) It is possible that NGOs’ estimates are collected from different populations with different underlying true values. NGOs often try to target lower wealth villages, and so baseline estimates may be worse off than the nationally representative DHS/MICS estimates. Note, however, that differences in household wealth indicators were small (e.g. “Household has electricity” 0.8% difference; “Household has a car” 0.2% difference). Additionally, the differences between DHS/MICS and NGO estimates might reflect actual changes over the years or across different geographical locations. Results from the analyses comparing data from the same source (DHS) but from different years and geographical levels also resulted in large differences between estimates.
(ii) Different methods employed while sampling, collecting, processing and analyzing data might also have contributed to the differences between DHS/MICS and NGO estimates.
(iii) Several indicators related to maternal and child health included in this study have not been validated and some have been shown to have low validity, such as maternal report of skilled birth attendance ( Blanc *et al.*, 2016). Inappropriate conflation of answer options and inconsistent coding and analysis of DHS surveys has also been documented ( Footman *et al.*, 2015). High measurement error can result in bias in unpredictable direction and dimension, resulting in large differences between estimates.

Whatever the cause of the large differences between estimates was, it was not possible to know which of the data sources (DHS/MICS or NGO) provided the most accurate estimation of the true prevalence in the NGOs target populations. Furthermore, while we have been comparing DHS/MICS and NGO point estimates, these indicators are measured with error. The standard error (SE) for the DHS indicators is greater than 5% in eleven percent of the estimates. An estimate with a standard error of 5% will have a 95% confidence interval of ± 9.8%.

Our analyses document and try to understand the large differences between NGO and DHS/MICS estimates. However, a study comparing DHS data to a small population-based survey from Rwanda showed that nine out of fifteen indicators related to maternal, newborn and child health were within a 10% difference ( Langston *et al.*, 2015). Similarly, in case studies from Nepal and Vietnam ( HealthBridge, 2020) there were many indicators where the DHS/MICS and NGO estimates were similar. In Nepal 70% of indicators were within 20% of one another. Estimates for ANC, iron-folic acid uptake, vitamin A supplementation at 18-23 months and mobile ownership were similar while breastfeeding, child dietary diversity and tetanus vaccination in pregnancy differed widely. In contrast, in Vietnam NGO estimates for exclusive/continued breastfeeding and dietary diversity at 6-8 months were close to DHS, while others differed by >30%. Using secondary data may be useful, especially in situations of budget or mobility restraint, such as during the COVID-19 pandemic with limited data collection opportunities. However, use of DHS surveys may risk underestimating the scale of problems for poor and marginalised groups such as nomads or slum dwellers ( Carr-Hill, 2017). When using DHS/MICS data, the user must keep in mind the potential differences between DHS/MICS and NGO estimates.

This study had some limitations. Most NGO data we used came from unpublished, not peer-reviewed reports created for internal use only. Indicators extracted from NGO reports were not necessarily consistent across all reports and often SDs or SEs were missing. Although, we matched the methods employed by the NGO as closely as possible in order to obtain the same indicators from DHS/MICS, some reports provided limited information concerning methods of data collection and analysis. Dates of and season of data collection were impossible to assess for eight reports. Assigning the geographical level of data from the NGO report was also challenging for some settings due to lack of contextual information. However, we were able to communicate with several NGOs in order to obtain supplementary information about the reports’ methods.

## Conclusion

Our hypothesis was that publicly available data can provide estimates of baseline conditions similar to those reported in NGO baseline reports when matched as closely as possible for location, year, and season of data collection. Our answer to this, in brief, is that publicly available data can be used, if the NGO is tolerant of imprecise estimates.

While an NGO may use the evidence presented here to justify forgoing their own baseline survey, they should keep in mind that DHS and MICS provide estimates for only some of the indicators of interest to the NGO. On average, we estimated 18 of the NGO’s indicators using DHS/MICS, but NGOs were often reporting 100+ estimates. Furthermore, collecting data in the NGO working area can provide valuable insights for project design and implementation.

## Data availability

This study used data owned by the DHS, the MICS and the NGOs that shared their baseline report. The DHS data can be downloaded at: https://www.dhsprogram.com, and the MICS data can be obtained at: https://mics.unicef.org. The DHS and MICS require registration and data access are only granted for legitimate research purposes.

The NGO reports were either available online on each NGO website or obtained by personal contact by email. The full list of NGO reports used in this study including report title, year of publication, organization name and how to access each report can be found at:

Harvard Dataverse: Details on reports used in the Maxdata project. https://doi.org/10.7910/DVN/32FUQV ( Berti, 2021).

Data are available under the terms of the Creative Commons Zero “No rights reserved” data waiver (CC0 1.0 Public domain dedication).
