# Supplementary material for: Using DHS and MICS data to complement or replace NGO baseline health data: an exploratory study
Source: F1000Res. 2021 Feb 4;10:69. [Version 1] doi: 10.12688/f1000research.47618.1 (PMC8145218; doi:10.12688/f1000research.47618.1)
Supplement: Peer review history

# Peer review: Reviewer response for version 1

**DOI:** 10.5256/f1000research.50696.r78936

**Published:** 2021-05-24

**Authors:** Neff Walker

**Affiliations:** 1 Senior Scientist, Johns Hopkins Bloomberg School of Public Health, Baltimore, MD, USA

This paper addresses a very interesting question but I must admit I remain unconvinced that the analyses presented here actually answer the question raised. From my perspective and that of the authors, the answer to this question is primarily related to how representative the data from the big surveys are of the program population. So for me, at one level the answer to this question is rather straightforward. If an NGO is working at provincial level and there has been a recent (in the last year) DHS or MICS survey that was sampled to be representative at the provincial level, then I would always use the DHS and MICS data as the baseline data forgoing the data collection by the NGO. Not only does this save time and energy, but my bias is that I believe the methods (e.g., sample size, mapping and household listing for sampling) used by major surveys like DHS and MICS are almost better than what an NGO would use. Of course, the answer to this question becomes less clear as the DHS or MICS survey data become less representative of the ideal program baseline, either in time or population. This is in large part what the analyses presented in this paper were all about.

 On the time issue, would I still use the DHS data if it was two years old? In large part the answer to that question depends on how rapidly the indicators you are measuring change. If, for example, one was interested in measuring baseline values such as total fertility rate, household composition, wealth which change very slowly two or even five year period between the survey and the start of the NGO program would not be a big concern. However, for indicators that may change quickly, say coverage of some interventions like bednet ownership, vitamin A supplementation coverage which can quickly be scaled up with campaigns, data from a survey that is two or more years old would probably not provide a very accurate measure. While the analyses presented here did some work to look at this issue I am not really sure that it was captured in their analyses.

 On population representation, using data from a very recent DHS survey that is sampled to be representative at a provincial level as baseline data for a program that covers 80% of the province may be okay. The data are not perfectly representative but unless there is extreme heterogeneity in the indicators of interest using the indicator values from the province probably provide a reasonable estimate of baseline coverage and therefore could replace a separate NGO-run survey. As with time, the key is how much variability is there within the population that was sampled for the DHS or MICS survey. This is hard to know, but clearly urban rural differences, ethnic mixes, topology all link to this. Again, I would think this would be a major issue that again, am not sure is captured in the current analyses. 

 I think these are exactly the issues that the authors were seeking to address in their analyses so why do I feel like these results do not really help us much in finding the answer? In their analyses they matched DHS/MICS data to data from NGO’s and then looked at how well these points matched when they varies in time or population. One major issue for me revolves around the NGO survey data. The analytical approach basically assumes that NGO surveys produced the right answer (as they are for the correct population and for the right time) ignoring that methodological or procedural weakness in the NGO surveys (e.g., lack of household listing, mapping during sampling, small sample size, poor training and supervision of interviewers) may make their results far from a gold standard comparison. I think I would be happier with analyses that restricted the comparisons to NGO surveys where a review of the methods and procedures and the sample size of the survey make me more confident about the quality of the NGO estimates.

 The second issue I have is the inclusion of data from DHS and MICS surveys that is broken down into smaller geographic region that was part of the sampling frame. Even if there are sufficient households at the district level, I am left wondering why we would expect the data to be very representative if that was not part of the sampling frame. I suppose that was part of the authors point of the analyses, but then we were not surprised that we found very weak correspondence between estimates of variables at the smaller geographic areas. I am a bit surprised the authors did not build a bit more on previous work on small area estimation that has tried to address many of the same issues focusing not just on differences due to population differences but also on techniques to adjust for these.

Is the work clearly and accurately presented and does it cite the current literature?

Partly

If applicable, is the statistical analysis and its interpretation appropriate?

Yes

Are all the source data underlying the results available to ensure full reproducibility?

Yes

Is the study design appropriate and is the work technically sound?

Partly

Are the conclusions drawn adequately supported by the results?

Yes

Are sufficient details of methods and analysis provided to allow replication by others?

Yes

Reviewer Expertise:

Modeling, program evaluation, estimation procedures

I confirm that I have read this submission and believe that I have an appropriate level of expertise to confirm that it is of an acceptable scientific standard.

# Peer review: Reviewer response for version 1

**DOI:** 10.5256/f1000research.50696.r78933

**Published:** 2021-03-16

**Authors:** Luay Basil

**Affiliations:** 1 Canadian Red Cross Society, Ottawa, ON, Canada

The paper addresses an important issue tackled frequently by NGOs that struggle to decide whether to use existing data or collect their own at baseline. It follows a rigorous methodology and uses a good number of studies to draw conclusions from. Thank you for the excellent work. Below are some reflections, questions and suggestions.

**Introduction:**

 Statement in p3 “In addition, the accuracy of the indicator estimates in NGO-led surveys

 may be insufficient for project design and monitoring purposes, due to relatively small sample sizes and the inherent high variability of the indicators of interest.”

- Since the statement is in the introduction section, and since the paper shows later that sampling errors are not a factor for the studies that met the criteria of inclusion, how do we reconcile between both?
- A sample size of NGOs might be adequate for indicators such as antenatal care, postnatal care, delivery by skilled birth attendant, but not for illnesses among children under 5 years when we ask about children with symptoms in the past 2 weeks or when measuring the prevalence of early child marriage among adolescents since the age group is between 14 and 17, especially if the focus is on girls. Have such indicators been part of the simulations to see the potential errors by NGOs in sampling? Can the paper mention whether sample size by NGOs is a factor in such cases? Are such indicators the one that are in the small error of sampling in Figure 3? If so, would it be better for NGOs to use DHS/MICS data even when the surveys are a few years old or for a higher level of geography compared to their areas?
- In addition to the challenges in sample size, sometimes the questionnaire in NGOs’ baseline surveys are not technically sound to measure a standard indicator. Has this been observed from the cases reviewed? If so, the paper could refer to that and add suggest using the questionnaire of DHS/MICS if the NGO is going to collect its own baseline data, especially since they are adjusted to local context.

**Table 4:**

 Scenario 1 shows improvement for a geography over time but there is not much difference within the provinces or geographies in the same year.

 Scenario 2 shows there is not much difference between 3rd level and national level in the same survey.

 Scenario 3 shows estimates of a later study that is not much different from earlier (slight improvement) because the earlier performance was quite high at 88.5%.

 Question: The table illustrate an important point; to what level is it representative of all examined studies? It would be good to mention that. If it is not representative, could the paper cite another example where there are significant differences? Or, at least mention that there is, if this is the case. See related comment on Table 4 later.

**Table 6:**

 Is there a need to explain in the methodology the rationale behind selecting 5% and 20% as the thresholds for comparison of differences? What would the picture be if the thresholds were 10% and 20%?

**Discussion:**

 P19 statement “In general, larger sample sizes were obtained at higher geographical levels and the larger the sample size (with their smaller sampling error) from DHS/MICS or NGO, the smaller the mean absolute difference between estimates. This meant that the advantage of geographical proximity is offset by the larger sampling error associated with small sample sizes.” One would expect that the NGO would calculate the adequate sample size required using a sample calculator (like the one of RADAR project). If the resources available would result in a sample size that is significantly less than the adequate one, should there be a recommendation that the NGO uses DHS/MICS data?

 P19 statement “It is possible that NGOs’ estimates are collected from different populations with different underlying true values. NGOs often try to target lower wealth villages, and so baseline estimates may be worse off than the nationally representative DHS/MICS estimates. Note, however, that differences in household wealth indicators were small (e.g. “Household has electricity” 0.8% difference; “Household has a car” 0.2% difference).” Since the DHS presents some findings by wealth quintiles, one would expect that findings from the lowest quintile could represent the areas NGOs work in and be close to those from NGOs data. Was comparison between indicators values from NGOs and the lowest wealth quintile from DHS made? If so, could you add a statement to reflect that?

 P 19 statement “Additionally, the differences between DHS/MICS and NGO estimates might reflect actual changes over the years or across different geographical locations. Results from the analyses comparing data from the same source (DHS) but from different years and geographical levels also resulted in large differences between estimates.” Table 4 for Zambia does not show large differences. Are there other studies that have that?  

**Conclusions:**

 P 22 “Our hypothesis was that publicly available data can provide estimates of baseline conditions similar to those reported in NGO baseline reports when matched as closely as possible for location, year, and season of data collection. Our answer to this, in brief, is that publicly available data can be used, if the NGO is tolerant of imprecise estimates.” The paper also shows that NGO can use DHS/MICS when the values of the indicators are very high or very low.

 P 22 statement “While an NGO may use the evidence presented here to justify forgoing their own baseline survey, they should keep in mind that DHS and MICS provide estimates for only some of the indicators of interest to the NGO. On average, we estimated 18 of the NGO’s indicators using DHS/MICS, but NGOs were often reporting 100+ estimates. Furthermore, collecting data in the NGO working area can provide valuable insights for project design and implementation.” It would be good to expand on this in the discussion section. NGOs’ need to measure different outcome levels on knowledge, attitudes and practice; the first two guide project design, implementation and setting targets. NGOs also need to report on the different outcome levels to their donors. DHS/MICS focus more on practice/utilization of services and less on attitude and knowledge. 

**Additional point**

 In a webinar presenting the paper in March 2, 2021, it was mentioned if an NGO wants to have a baseline so it can compare with the end-line, it can have a properly randomized and controlled end-line that can give good findings on the project’s impact. Could that be added to the paper?

Is the work clearly and accurately presented and does it cite the current literature?

Yes

If applicable, is the statistical analysis and its interpretation appropriate?

I cannot comment. A qualified statistician is required.

Are all the source data underlying the results available to ensure full reproducibility?

Yes

Is the study design appropriate and is the work technically sound?

Yes

Are the conclusions drawn adequately supported by the results?

Yes

Are sufficient details of methods and analysis provided to allow replication by others?

Yes

Reviewer Expertise:

I have been involved in several baseline and end-line surveys for projects in pubic health conducted in developing countries and have witnessed the pros and cons of conducting them. My engagement has been in the planning, design, data analysis and reporting phases. I have also used findings from the DHS/MICS to guide project design, implementation and evaluation.

I confirm that I have read this submission and believe that I have an appropriate level of expertise to confirm that it is of an acceptable scientific standard.
